# Lung endothelial cytopathic tau is sufficient to impair long-term potentiation during infection

**DOI:** 10.1093/ajrcmb/aanag040

**Published:** 2026-03-10

**Authors:** Mike T Lin, Mikhail Alexeyev, Jakob Körbelin, Chun Zhou, Samantha D Chaney, Chung-Sik Choi, Charu Shastri, Hector D Chavarria-Bernal, Nancy H Chen, Madeline Stone, Allison J Bauman, Linn Ayers, Reece P Stevens, Ji Young Lee, Sarah L Sayner, Ron Balczon, Amy R Nelson, Troy Stevens

**Affiliations:** Department of Physiology and Cell Biology, Center for Lung Biology, University of South Alabama, Mobile, AL, United States; Department of Physiology and Cell Biology, Center for Lung Biology, University of South Alabama, Mobile, AL, United States; Department of Oncology, Hematology, and Bone Marrow Transplantation, University Medical Center Hamburg-Eppendorf, Hamburg, Germany; Department of Physiology and Cell Biology, Center for Lung Biology, University of South Alabama, Mobile, AL, United States; Department of Molecular Pharmacology and Physiology, University of South Florida, Tampa, FL, United States; Department of Physiology and Cell Biology, Center for Lung Biology, University of South Alabama, Mobile, AL, United States; Department of Pathology, University of South Alabama, Mobile, AL, United States; Department of Pathology, University of South Alabama, Mobile, AL, United States; Department of Physiology and Cell Biology, Center for Lung Biology, University of South Alabama, Mobile, AL, United States; Department of Physiology and Cell Biology, Center for Lung Biology, University of South Alabama, Mobile, AL, United States; Department of Physiology and Cell Biology, Center for Lung Biology, University of South Alabama, Mobile, AL, United States; Department of Physiology and Cell Biology, Center for Lung Biology, University of South Alabama, Mobile, AL, United States; Division of Pulmonary and Critical Care, Department of Medicine, Johns Hopkins University School of Medicine, Baltimore, MD, United States; Departments of Physiology and Cell Biology and Internal Medicine, Center for Lung Biology, University of South Alabama, Mobile, AL, United States; Department of Physiology and Cell Biology, Center for Lung Biology, University of South Alabama, Mobile, AL, United States; Department of Biochemistry and Molecular Biology, Center for Lung Biology, University of South Alabama, Mobile, AL, United States; Department of Molecular Pharmacology and Physiology, University of South Florida, Tampa, FL, United States; Departments of Physiology and Cell Biology and Internal Medicine, Center for Lung Biology, University of South Alabama, Mobile, AL, United States

**Keywords:** *Pseudomonas aeruginosa*, acute respiratory distress syndrome, blood-brain barrier, hippocampus, long-term potentiation

## Abstract

Cytopathic tau variants are recovered from the lung, circulation, and brain following lower respiratory tract infection. Cytopathic tau injures the lung and brain, yet its cellular origin during infection is unknown. Here, we assessed whether lung capillary endothelium is a source of cytopathic tau that contributes to brain injury during infection. Alveolar-capillary permeability was higher in tau knockout than wild-type mice following sublethal *Pseudomonas aeruginosa* infection, indicating that endogenously expressed tau contributes to integrity of the lung’s gas exchange unit. Hippocampal long-term potentiation was inhibited following sublethal infection in wild-type but not tau knockout mice, even though the blood-brain barrier was not overtly disrupted. Tau expression solely in lung capillaries of tau knockout mice was sufficient to restore alveolar-capillary barrier integrity and impair hippocampal long-term potentiation following sublethal infection. Thus, endogenous lung capillary endothelial tau preserves alveolar-capillary integrity, yet it is a source of cytopathic tau that injures the brain during pneumonia.

Clinical Relevance Lower respiratory tract infection triggers host responses that contribute to end-organ dysfunction. Cytopathic tau variants are generated during infection and have been shown to injure both the lung and brain, yet the cellular origin of injurious tau variants is unresolved. Here, we demonstrate that *P. aeruginosa* pneumonia elicits generation of cytopathic tau in lung endothelium, and the endothelial-derived tau accesses the brain where it inhibits hippocampal long-term potentiation.

## Introduction

Lower respiratory tract infection is an incident cause of impaired learning and memory.^1^ This phenomenon has been reported in patients with community-acquired pneumonia who are not critically ill,^2^ and it has been seen in patients with ventilator-associated pneumonia, the acute respiratory distress syndrome, and sepsis who are critically ill.^3-6^ In the latter cases, delirium in the intensive care unit portends susceptibility to and severity of the protracted cognitive impairment.^7^^,^^8^ Approximately one-third of patients exhibit mild to severe forms of cognitive impairment 1–2 years after their release from the intensive care unit.^6^ An even higher proportion of patients report anxiety, depression, posttraumatic stress syndrome, and poor neuromuscular strength, balance, and coordination.^1^^,^^4^^,^^6^^,^^9-11^ The mechanism(s) of these incident forms of neurological impairments is unknown.

In some instances, infection promotes systemic inflammation that can also cause neuroinflammation.^12^^,^^13^ However, it is not clear whether, or exactly how, the systemic cytokine response is associated with cognitive impairment.^14^ In other cases, infection causes hypoxemia, and hypoxemia injures the brain.^4^^,^^15^ Yet, hypoxemia is not present in many patients with infection who exhibit cognitive impairment. Recently, 2 different mechanisms have been proposed to link either pneumonia or sepsis with breakdown of the blood-brain barrier, neuroinflammation, and/or impaired neural information processing in the hippocampus. Hippensteel and colleagues^16^^,^^17^ reported that sepsis degrades the endothelial cell glycocalyx and releases 2-*O*- and *N*-sulfated heparan sulfate fragments that are translocated from the blood into the brain in association with blood-brain barrier breakdown, where they impair hippocampal long-term potentiation. Alternatively, lower respiratory tract infection leads to generation of cytopathic tau variants that are recovered from the airways, blood, and the brain; the infection-elicited cytopathic tau inhibits hippocampal long-term potentiation.^18-22^ Whether these 2 mechanisms independently contribute to impaired neural information processing during infection or whether they are mechanistically linked remains unclear. Nonetheless, in the latter example, the origin of cytopathic tau during infection is a major point of controversy.

The lung itself may be a source of cytopathic tau during infection. The lung atlas reveals that at least 4 nonneuronal cell types within the lung express tau, including adventitial fibroblasts, pericytes, lymphatic endothelium, and vascular endothelium.^19^^,^^23^ The highest tau-expressing endothelia are found in capillaries, which are comprised of 2 phenotypically distinct cell types, the general capillary (Cap1) and aerocyte (Cap2) endothelia.^24^^,^^25^ Both general capillary and aerocyte endothelial cells express tau.^19^^,^^23^^,^^24^ Capillary endothelium is closely juxtaposed to type I alveolar epithelium, and together, these cell types form the alveolar-capillary membrane necessary to optimize gas exchange. Capillary endothelium senses and responds to the microenvironments within the alveolus and the blood. The contribution of endogenously expressed tau to alveolar-capillary barrier integrity is unknown. Moreover, whether global tau expression is necessary, and more specifically, whether capillary endothelial tau expression is sufficient for infection to impair hippocampal neural information processing is unresolved. We found that lung capillary endothelial tau expression not only contributes to integrity of the alveolar-capillary membrane, it is also a source of cytopathic tau that impairs hippocampal neural information processing during lower respiratory tract infection. Some of the results of these studies have been previously reported in abstract form.^26^

## Materials and methods

### Mice and bacteria

Experimentation with animals was approved by the Institutional Animal Care and Use Committee of the University of South Alabama, and conducted according to the “Guide to the Care and Use of Laboratory Animals.” Adult wild-type (C57BL/6J) and tau knockout (B6.129S4(Cg)-*Mapt*^tm1(EGFP/Klt^/J, Stock # 029219, The Jackson Laboratory, Bar Harbor, ME, USAs) mice of both genders were used. For primary infection studies, mice were anesthetized by 1.5% isoflurane and inoculated intratracheally with the *Pseudomonas aeruginosa* (PA103Δ*exoUexoT*::Tc pUCP*exoY*; 1 × 10^5—^5 × 10^5^ colony-forming units [CFU] in 40 µL). The PA103Δ*exoUexoT*::Tc pUCP*exoY* mutant is derived from PA103, yet exoenzymes U and T have been removed and exoenzyme Y has been introduced via a plasmid.^18^^,^^21^^,^^22^ The resulting mutant possesses a functional type III secretion system and introduces only exoenzyme Y into host cells. The bacteria were taken from frozen stocks and grown overnight on solid agar/carbenicillin (400 µg/mL) media. Bacteria were resuspended in phosphate-buffered saline (PBS) to an OD_540_ of 0.25, which has previously been determined to equal 2 × 10^8^ bacteria/mL. Dilutions were made in PBS to attain the reported concentration. At 48 hours after the infection, mouse lungs, hippocampus, cortex, heart, kidneys, liver, spleen, and plasma (∼0.5-1 mL) were collected under isoflurane anesthesia or immediately after euthanasia and snap frozen in liquid nitrogen.

### Ultrasound lung imaging

A Vevo 3100 (VisualSonics, Toronto, ON, Canada) with a 15-30 MHz MX250 transducer was used to assess lung fields. Spontaneously breathing mice were anesthetized using 1.5%-2% isoflurane (titrated as needed) in a 1:1 O_2_-air admixture. Heart rate, electrocardiogram, and respiration were continuously recorded using a sensor-embedded exam pad. All measurements were made within a 20-minute time period in order to minimize the potential for cardiovascular stress, as described previously in detail.^18^^,^^27^

### AAV production

AAV particles were produced as described previously,^28^^,^^29^ following the Körbelin lab protocol with minor modifications. The cDNA encoding an N-terminally 3×FLAG-tagged, codon-optimized version of the mouse tau isoform 1N4R was commercially synthesized by back-translating the amino acid sequence and cloned into either the pAAV-MCS vector (Stratagene), generating the intron-containing plasmid pMA6078, or into pAAV-CMV-eGFP (replacing eGFP), generating the intronless plasmid pMA6082. The pAAV-CMV-eGFP vector was derived from pAAV-CMV-Luc.^30^ For AAV production, 293 cells were transfected with an equimolar mixture of 3 plasmids: (1) pMA6078 or pMA6082; (2) pHelper (Stratgene); and (3) pXX2-187-ESGHYF, which encodes AAV2 rep and cap genes, with the cap gene modified for lung endothelium targeting.^28^ Per 150-mm dish, 50 µg of total DNA and 150 µg of linear polyethyleneimine (average molecular weight ∼25 kDa; Polysciences) was used, with 20 to 30 dishes per production batch. The culture medium was changed 18 hours after transfection and collected at 72 and 120 hours. Both collected medium^31^ and cell pellets were processed for AAV purification using step iodixanol gradient ultracentrifugation.^32^ The iodixanol-purified AAV particles were subsequently concentrated and buffer-exchanged into PBS containing 0.001% Pluronic F-68 using Amicon Ultra-15 centrifugal filters (100 kDa molecular weight cut-off; Sigma). The final viral genome titer was determined by digital droplet PCR.^33^

### Isolated perfused lung

Wild-type and tau knockout mice were anesthetized using pentobarbital sodium (100 mg/kg body weight in Fatal Plus solution). Once a surgical plane was achieved, as defined by the absence of a withdrawal reflex following toe and tail pinch, mice were intubated and ventilated, a sternotomy was performed, and pulmonary artery and left ventricle/atrium catheters were placed. Heart and lungs were removed en bloc and suspended in a humidified chamber, where mechanical ventilation and flow were established. Mice lungs were perfused with buffer (in mmol/L: 119.0 NaCl, 4.7 KCl, 1.17 MgSO_4_, 1.18 KH_2_PO_4_, 23 NaHCO_3_, and 5.5 glucose) containing 4% bovine serum albumin and physiological (2.2 mmol/L) CaCl_2_. Lungs were perfused at 2 mL/minute in either anterograde or retrograde orientation for 20 minutes to obtain an isogravimetric state, and then flow was increased by 2 mL/minute every 5 minutes.^34^ Pulmonary artery and venous pressures and lung weight were measured continuously, and double occlusion pressure was measured at the end of each 5-minute interval. Data were recorded by the PowerLab system and analyzed with LabChart 8 data analysis software from AD Instruments.

In studies testing the permeability-evoking effect of lipopolysaccharide (LPS; from *Escherichia coli* O111:B4, Sigma-Aldrich), LPS (40 µL) was added to the airways and the perfusate, after the heart and lungs were isolated and ventilated. LPS was allowed to recirculate for 2 hours under isogravimetric conditions, prior to initiating increases in perfusion rates, as described above.

### Anterograde and retrograde lung perfusion

Stopcocks and a bypass circuit were arranged to immediately reverse flow direction from the forward to reverse orientations.^34^ This switch permitted uninterrupted flow via the perfusion pump, through the bubble trap, heat exchanger, and inflow and outflow reservoirs. The lung did not experience intermittent flow in this circuit.

Flow was first established in the forward orientation. After an isogravimetric period, flow was reversed without disconnecting the circuit, by directing perfusate through 2 cross-connections between the pulmonary artery and left atrial lines. Lungs were perfused with increasing flow starting from 2 mL/minute, and lung weight and hemodynamic parameters were continuously assessed as described. Filtration coefficient (K_f_) was determined by measuring the rate of lung weight gain in response to an increase in double occlusion pressure from elevated flow rates, relative to double occlusion pressure at the baseline flow rate of 2 mL/minute, and normalized to 100 g of predicted wet lung weight.

### Tau measurement in the plasma

Animals were anesthetized using 2% to 3% isoflurane and the ribs were dissected to expose the heart. A tuberculin syringe with a 25-gauge needle was inserted into the right ventricle and blood was slowly drawn to collect approximately 0.5 mL in heparin (0.05 µL). Whole blood was centrifuged at 200*g* at 4 °C for 10 minutes (Eppendorf 5910 R). Once separated, the plasma was removed and stored at −80 °C until further use.

Tau was measured in the plasma using the MSD R-PLEX mouse total tau assay (#K1528ER-2, Kit Lot #511510) with SECTOR plates. Tau was measured in 25 µL of undiluted plasma, and each sample was run in duplicate. This assay employs an electrochemiluminescence detection method to quantify total tau protein, in accordance with the manufacturer’s instructions. Plasma tau concentrations were determined from the MSD calibration curve, with a recommended lower limit of ∼100 pg/mL and the sensitivity range extending to ∼40 pg/mL. The average coefficient of variation was less than 4% for samples.

### Transcardial perfusion

The mice were anesthetized with a ketamine/xylazine mixture (100/5 mg/kg of body weight) via intraperitoneal injection 48 hours postinfection. They were then transcardially perfused with 35-45 mL PBS (#P3813, Sigma-Aldrich, St. Louis, MO, USA) with 5 mM EDTA at a rate of 5 mL/minute. One brain hemisphere was snap frozen on dry ice or in liquid nitrogen and the other hemisphere was embedded in optimal cutting temperature (#4583, Sakura, Torrance, CA, USA) compound. The embedded hemisphere was used for serial tissue cryosectioning at 18 μm thickness, with 4 sections of tissue about 270 μm apart mounted on each microscope slide and stored at −80°C.

### Lung histology and brain immunohistochemistry

Lung histology was assessed 24 and 48 hours following intratracheal delivery of sublethal *P. aeruginosa* (1 × 10^5^ CFU in 40 µL). Wild-type and tau knockout mice were anesthetized, their chests were retracted, and pulmonary artery and left atrial catheters were placed. Blood was cleared using 5 mL of PBS at inflow and outflow pressures of 25 cm H_2_O and 5 cm H_2_O, respectively. Formalin was perfused through the circulation with the same pressures and then catheters were clamped to retain vascular pressure. A tracheal tube was placed and the airways were filled with 700 µL of formalin, representing approximately 65% of total lung capacity. The heart and lungs were dunked in formalin, while maintaining vascular and airway pressures. Paraffin-embedded lung blocks were sectioned across injured areas of multiple lung lobes, including the left lung and the right upper, middle, and lower lobes. A heterogeneous pattern of pneumonia was seen in this sublethal infection (ie, 100% survival to the time of the terminal surgery). A total of 4 lung regions were represented in the analyses, each with 6 to 8 serial sections. In total, 48 slides were analyzed, representing sections obtained from 3 animals (2 male and 1 female) at each time point, in wild-type and tau knockout animals.

Lung sections were analyzed by 2 pathologists in a blinded fashion. Consensus was achieved in scoring among the reviewers to determine a cumulative lung injury score, based on accepted scoring criteria.^35^ In short, 5 scoring areas were evaluated, including evidence for (A) the presence of neutrophils in the alveolar space; (B) the presence of neutrophils in the interstitial space; (C) hyaline membranes; (D) proteinaceous debris filling the airspaces; and (E) alveolar septal thickening. The scoring range was 0 to 2, where 0 = none; 1 = 1 to 5; and 2 = more than 5. Scoring was determined from the following equation, where the total lung injury score = [(20 × A) (14 × B) (7 × C) (7 × D) (2 × E)]. Because the same number of lung images were taken across all genotypes and treatments, the total lung injury score is reported, and numerical values were not standardized to lung slices. Areas of atelectasis and evidence of hemorrhage were visible in lung sections and were quantitatively assessed, yet were not a part of the overall lung injury score.

In brain immunohistochemistry studies, following 10 minutes of fixation with 4% paraformaldehyde (#157-4, Electron Microscopy Sciences, Hartfield, PA, USA), the brain tissue sections were then washed 3 times with PBS for 5 minutes each wash. IgG, caldesmon, and glial fibrillary acidic protein (GFAP) staining and reagents were used; see Table S1 for additional details. In brief, tissue sections were incubated overnight at 4 °C with primary antibodies in blocking buffer (1% normal donkey serum [#S30-M, Sigma-Aldrich], 0.25% Triton X-100 [#X100], in 1× PBS). Slides were then subjected to 5-minute washes with PBS 3 times. Next, the slides were incubated at room temperature for 1.5 hours with secondary antibodies in blocking buffer. A conjugated lectin was added with the secondary antibodies to visualize blood vessels with the IgG and caldesmon slide sets. After 3 washes with PBS for 5 minutes each wash, the sections were cover slipped with DAPI Fluoromount-G (#17984-24, Electron Microscopy Services). Three randomly selected fields from each brain region (eg, hippocampus and cortex) from each of the 4 sections of tissue/animal were imaged on an ECHO Revolve microscope (#RVL-100-M, ECHO, San Diego, CA, USA) using an Olympus UPlanFL N, 20×/0.50 Ph1 fluorescence objective (Olympus, Tokyo, Japan).

### Blood-brain barrier permeability

IgG (150 kDa) leakage out of the vessels was visualized in association with a fluorescently labeled lectin. Vessels that were visualized with lectin staining and IgG were quantified by using the ImageJ Integrated Density measurement.

### Pericyte coverage

Caldesmon was used to visualize pericytes and was co-stained with the conjugated lectin. Following automated thresholding in ImageJ to separately analyze the signals from pericytes and vessels, their respective signals were measured using the ImageJ Integrated Density. Pericyte coverage was quantified as a percentage (%) of caldesmon-positive pericyte surface area covering lectin-positive capillary surface area per field.

### Multiphoton imaging

Precision lung slices were prepared using the agarose/gelatin-infused lung slice technique. Mice were sedated with injection of pentobarbital sodium intraperitoneally, and an anesthetic plane was verified by lack of toe pinch response. Mice received retro-orbital injection of *Lycopersicon esculentum* (tomato) lectin (Vector Labs, either DyLight 488, 594, or 649 to differ from the AAV reporter being used) to fluorescently label the endothelium. Mice were then injected with 0.1 mL of heparin intraperitoneally, and following thoracotomy the pulmonary artery was cannulated through the right ventricle. The left ventricle was nicked, and ∼10 mL of Hanks buffered saline solution (Gibco) was slowly perfused through the pulmonary circulation at an inflow pressure of 25 cm H_2_O and an outflow pressure of 5 cm H_2_O. Next, ∼10 mL gelatin (6%; Thermo Fisher Scientific) solution was perfused into the pulmonary circulation. The right lung was then sutured at the hilum, and a tracheal tube was inserted, followed by infusion of ∼1 mL of agarose (2.7%; Thermo Fisher Scientific) solution to inflate the lung. Ice was used in situ to solidify the gel en bloc. The left lung was sectioned into approximately 800-μm-thick serial sections using a vibratome. Slices were added to a 6-well, glass-bottom plate (Cellvis) and placed in an incubator for 1 hour or until imaging. Other tissues, including heart, kidney, and brain, were harvested and placed in buffered saline solution. Tissues were then cut into thin (∼0.5 mm) slices prior to imaging.

Imaging was performed using a Nikon A1R multiphoton microscope with a 20× objective, coupled to a dual-line Ti:sapphire laser (Insight DS , Spectra Physics) with 4 gallium arsenide phosphide detectors for simultaneous acquisition of multiple fluorophores. Both large image areas and z-stacks were acquired to visualize an entire section of the lung lobe and high-resolution images of AAV-driven fluorescence, as well as *Lycopersicon esculentum* (tomato) lectin and tau knockout enhanced green fluorescent protein (EGFP) signals.

### Western blotting

Tissue lysates were prepared by adding frozen tissues directly into a tube containing ice-cold RIPA buffer with 2 mM EDTA and 1× Holt protease and phosphatase inhibitor cocktail (Thermo Scientific, #1861281), followed by homogenization using a bead mill 24 homogenizer (Fischer Scientific, #15-340-163). After centrifugation at 14 500 RPM at 4 °C for 15 minutes, the supernatants were collected and the protein concentrations were determined. Samples were heated for 5 minutes at 95 °C and spun briefly prior to loading on a 4%-12% Bis-Tris gel for sample resolution. Proteins were transferred from the gel to a nitrocellulose membrane and probed with a FLAG M2 antibody (1:2000; Sigma-Aldrich, #F3165), tau 2E9 antibody (1:2000; Novusbio, #NBP2-25162), or actin antibody (1:4000; BD Biosciences, #612656). Blocking and incubation with antibodies were done in 6% nonfat milk–Tris-Buffered Saline with Tween 20. The secondary antibody for the FLAG M2, 2E9, and actin primary antibodies was the HRP-conjugated goat anti-mouse IgG (Invitrogen, #A16078) or the IgG light chain–specific antibody (Jackson ImmunoResearch, #115-035-174), each at 1:10 000.

### Hippocampal slice preparation

Hippocampal slices were prepared from mice that were 10–16 weeks old. Immediately upon euthanasia, the brain was quickly obtained and placed into ice-cold sucrose-artificial cerebrospinal fluid (CSF) (in mM): 70 sucrose, 80 NaCl, 2.5 KCl, 21.4 NaHCO_3_, 1.25 NaH_2_PO_4_, 0.5 CaCl_2_, 7 MgCl_2_, 1.3 ascorbic acid, and 20 glucose. Hippocampi were dissected, placed onto an agar block, and mounted on a Leica VT1200s (Leica Instruments) slicing chamber. Transverse hippocampal slices were cut at 300 µm thickness in sucrose-artificial CSF, and immediately transferred into a holding chamber containing regular artificial CSF (in mM): 125 NaCl, 2.5 KCl, 21.5 NaHCO_3_, 1.25 NaH_2_PO_4_, 2.0 CaCl_2_, 1.0 MgCl_2_, and 15 glucose. Slices were incubated at 35 °C for 30 minutes and then at room temperature for >1 hour before recording. All solutions were constantly equilibrated with 95% O_2_ and 5% CO_2_.

### Electrophysiology recordings

Experiments were performed at room temperature. Hippocampal slices were visualized using a fixed-stage upright microscope (Leica) equipped with infrared differential interference contrast optics. The recording chamber was continuously superfused with artificial CSF, containing SR95531 (2 µM) and CGP55845 (1 µM) to block inhibitory synaptic transmission, and flowing at 1 mL/minute. Both recording and stimulating electrodes were pulled from borosilicate pipettes (BF150-86-10; Sutter Instruments). CA3 region was severed to eliminate recurrent excitation; Schaffer collateral stimulation was achieved using an ISO-Flex stimulus isolation unit (A.M.P.I.) with stimulus duration of 0.1 ms. Recordings were obtained using an EPC10-dual amplifier (HEKA). Analog signals were further amplified 10× and filtered at 5 kHz using an Axopatch amplifier (Axon Instrument) and digitized at 20 kHz using Patchmaster software (HEKA). Evoked field excitatory postsynaptic potentials (fEPSPs) were recorded every 20 seconds. Following >10 minutes stable fEPSP baseline, a theta burst stimulation was delivered to induce synaptic strengthening. Long-term potentiation was calculated from 60 minutes poststimulation normalized to the baseline.

Offline data analysis and statistical comparisons were performed using custom macros written in Igor Pro (WaveMatrics). The fEPSP slope was measured between 20% and 60% of the rising phase. Data were binned at 3-minute intervals to generate summary fEPSP slope plots. Data are expressed as mean ± SD and compared statically using ANOVA followed by Tukey post hoc tests or using a nonparametric Wilcoxon–Mann–Whitney rank test. *P* <.05 was denoted as statistically significant.

### Statistical analysis

Quantitative data are presented as means ± SD or SEM, as described. Group size for all figures ranged from *n* = 4 to *n* = 5. Group means were compared using 2-way ANOVA with a post hoc test as appropriate. *P* <.05 was considered statistically significant.

## Results

### Endogenously expressed lung tau stabilizes the alveolar-capillary barrier during infection

Adaptation of the pulmonary circulation to increased blood flow and integrity of the alveolar-capillary barrier was assessed in wild-type and tau knockout mice. The pulmonary circulation accommodates anterograde (forward) and retrograde (reverse) blood flow equally well.^34^ Establishing the flow-pressure response to forward and reverse flow, respectively, provides insight into how each of the vascular segment(s) are impacted by infection and/or vascular remodeling.^34^ The isolated perfused lungs from wild-type and tau knockout mice exhibited similar pressure and permeability responses to both forward and reverse flow (Figure S1). The principal site of vascular resistance in each flow orientation was upstream of the capillary bed. The circulations of these mouse genotypes accommodated up to 10 mL/minute of flow, while maintaining the capillary pressure below the threshold (25 mm Hg or 33 cm H_2_O) that causes transudative edema.^36^ At 12 mL/minute of flow, capillary pressures increased above this threshold and the lung acutely developed edema requiring termination of the study. Thus, wild-type and tau knockout mice exhibit similar pulmonary vascular pressure responses to increases in perfusion rates, in both anterograde and retrograde flow orientations.

**Figure 1:**
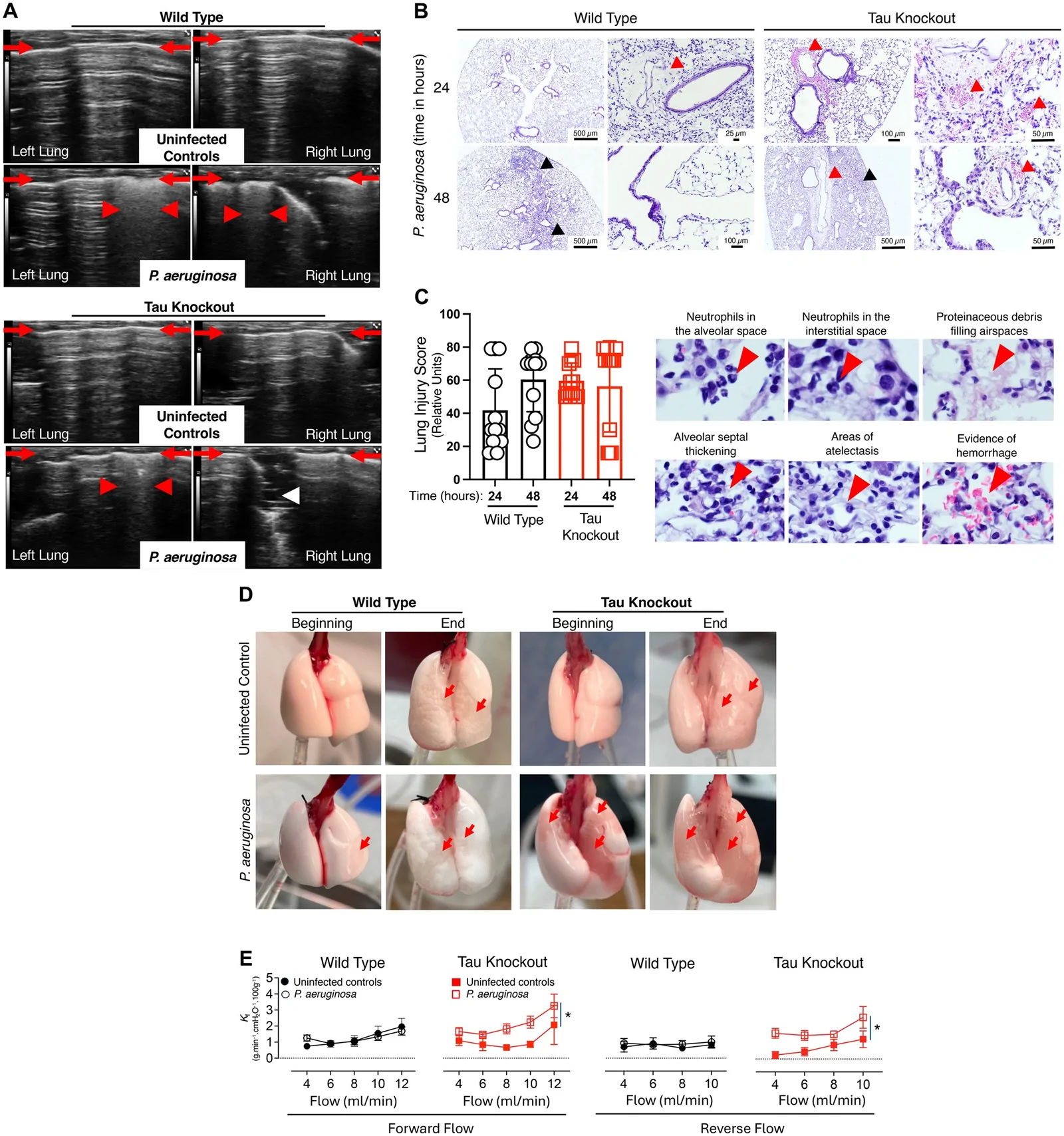
Endogenously expressed tau protects the alveolar-capillary membrane following bacterial infection. (A) Intratracheal inoculation of *P. aeruginosa* leads to a heterogeneous pattern of lung edema and consolidation 48 hours postinfection. Representative ultrasound images of the left and right lung fields. Uninfected controls display uniform A lines, characterized by hyperechoic regularly spaced horizontal repetitive lines, representing open and air-filled alveoli. Infected animals exhibited B lines, characterized by irregular focal hyperechoic opacities, representing fluid-filled airways with consolidation. Red arrows represent the pleural line, red arrowheads indicate B lines, and white arrowheads represent areas of consolidation. (B) Analysis of H&E-stained lung sections revealed a heterogenous pattern of lung injury in both wild-type and tau knockout mice 24 and 48 hours after infection. Inflammatory cell recruitment into the lung tissues and airways was evident in both groups (black arrowheads). Perivascular fluid cuffs were infrequently seen in lungs from wild-type animals, but were frequently visible in lungs from tau knockout animals (red arrowheads). Red blood cells were visible in nonvascular compartments, including in perivascular spaces and alveoli (red arrowheads). (C) Lung injury was quantified according to previously established standards, as described in the Materials and Methods.^35^ The total lung injury score was not different among 24- and 48-hour time points and was not significant between wild-type and tau knockout mice (*P* = not significant by one- and 2-way ANOVA). Examples of lung injury criteria are shown, highlighted by red arrowheads; hyaline membranes were not seen. (D) Lung tau expression improves alveolar-capillary barrier integrity following infection. Heart and lungs were removed en bloc. Lungs were ventilated and the circulation perfused over a range of flow rates (see also E). Representative lung images from uninfected control and infected mice are presented at the baseline perfusion rate of 2 mL/minute, and at the maximum flow rate achieved during the retrograde perfusion experiments. Lung injury was more prominent in the lungs from tau knockout mice. (E-G) Lung tau is an important determinant of the integrity of the alveolar-capillary barrier following infection. Perfusion rates were increased over time while pulmonary artery (P_PA_) and pulmonary vein (P_PV_) pressures were measured continuously and double occlusion (P_DO_) pressure was measured under stop-flow conditions. Permeability was assessed at each flow rate by filtration coefficient (K_f_). Although vascular pressures were similar in the lungs from wild-type and tau knockout mice, K_f_ was elevated in tau knockout mice, in both forward (anterograde; E, F) and reverse (retrograde; E, G) flow orientations, after *P. aeruginosa* infection. *n* = 5-6 mice per group. (H) Lungs from tau knockout mice are more susceptible to injury induced by lipopolysaccharide (LPS) than are lungs from wild-type mice. After lung isolation, perfusion, and ventilation, LPS (40 µL) was delivered to the airway and circulation. LPS was allowed to recirculate for 2 hours before the perfusion rate was increased and permeability was measured. Data are shown as mean ± SEM. *Significant difference, with *P* <.05, as assessed by repeated measures.

We then assessed the pulmonary circulation in wild-type and tau knockout mice 48 hours following sublethal bacterial infection. Mice infected with sublethal *P. aeruginosa* (ie, LD_0_) exhibited intermittent hyperechoic vertical B lines and consolidation in both lung fields as assessed by ultrasonography, suggestive of mild alveolar edema^18^ (Figure 1A). Histological analyses were also suggestive of a diffuse pattern of lung injury, with large unaffected lung regions aside inflammatory lung regions (Figure 1B). The involved areas exhibited a neutrophil-predominant recruitment into the tissue and alveoli (Figure 1C). Lungs from wild-type and tau knockout mice displayed evidence of fluid accumulation. Lungs from tau knockout mice exhibited extensive perivascular cuffing with accumulation of red blood cells in the perivascular spaces as well as in the alveoli. Altogether, histological assessment revealed that similar levels of lung injury were present in wild-type and tau knockout mice, yet a hyperpermeable response to infection was evident in the tau knockout mice.

**Figure:**
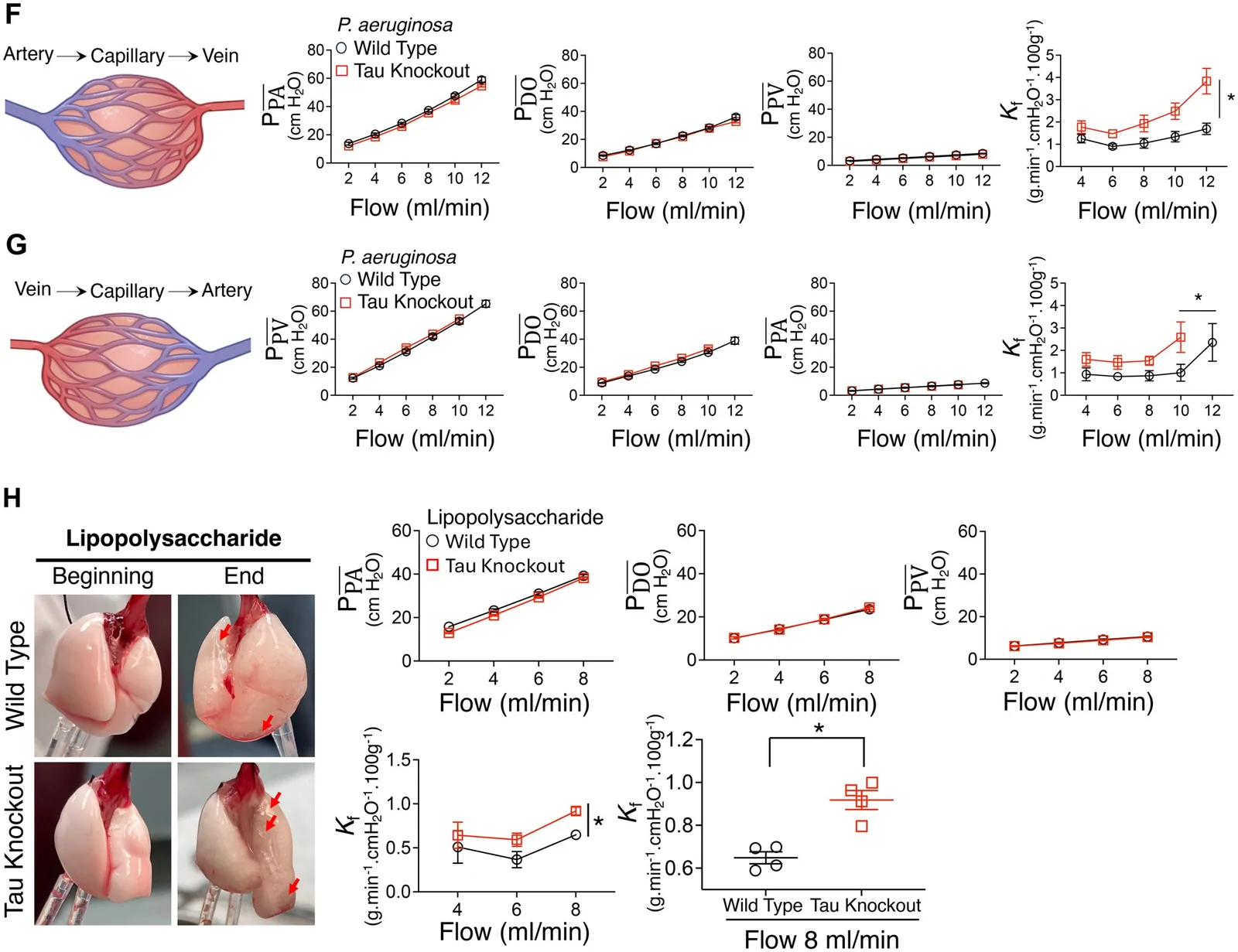


Whereas the lungs from wild-type mice did not appear injured from bacterial infection by gross inspection following isolation and perfusion, a heterogenous pattern of lung injury was evident in the tau knockout mice (Figure 1D). Gross morphology of the lungs after perfusion at high flows illustrated a more pronounced disruption of endothelial barrier integrity in tau knockout mice, whereas lungs from wild-type mice exhibited a less severe impact of increased flow after infection (Figure 1E). Pulmonary artery, capillary (ie, double occlusion pressure), and vein pressures were similar in lungs of wild-type and tau knockout mice in response to increased perfusion flow rates, yet tau knockout mice exhibited a greater sensitivity to disruption of the alveolar-capillary barrier integrity following infection (Figure 1F, G). The pulmonary circulation of wild-type mice accommodated increases in flow up to 12 mL/minute before developing pulmonary edema. In contrast, alveolar-capillary membrane permeability was higher in tau knockout mice across all perfusion rates, in both anterograde and retrograde perfusion orientations (Figure 1E). The pulmonary circulation of tau knockout mice did not accommodate flows >10 mL/minute, even though capillary pressures were below 25 mm Hg, consistent with exudative or permeability edema. These findings indicate lung capillary endothelium is more susceptible to injury in the tau knockout mice than in wild-type controls following sublethal bacterial pneumonia.

To assess whether the alveolar-capillary barrier in tau knockout mice is more sensitive to a direct inflammatory insult than in wild-type mice, a low concentration of LPS was administered to both the airways and circulation in the isolated perfused lung. LPS was allowed to recirculate for 2 hours under isogravimetric conditions, before increasing perfusion rates and assessing permeability. Exposure to this low LPS concentration did not significantly increase permeability in lungs from wild-type mice, yet permeability was significantly higher in lungs from tau knockout mice (Figure 1H). Neither the pulmonary artery, double occlusion, nor venous pressures were different among groups. Thus, tau contributes to alveolar-capillary barrier integrity, during bacterial infection in vivo and upon exposure to LPS, in the absence of bacteria.

### Sublethal infection does not overtly disrupt the blood-brain barrier

**Figure 2:**
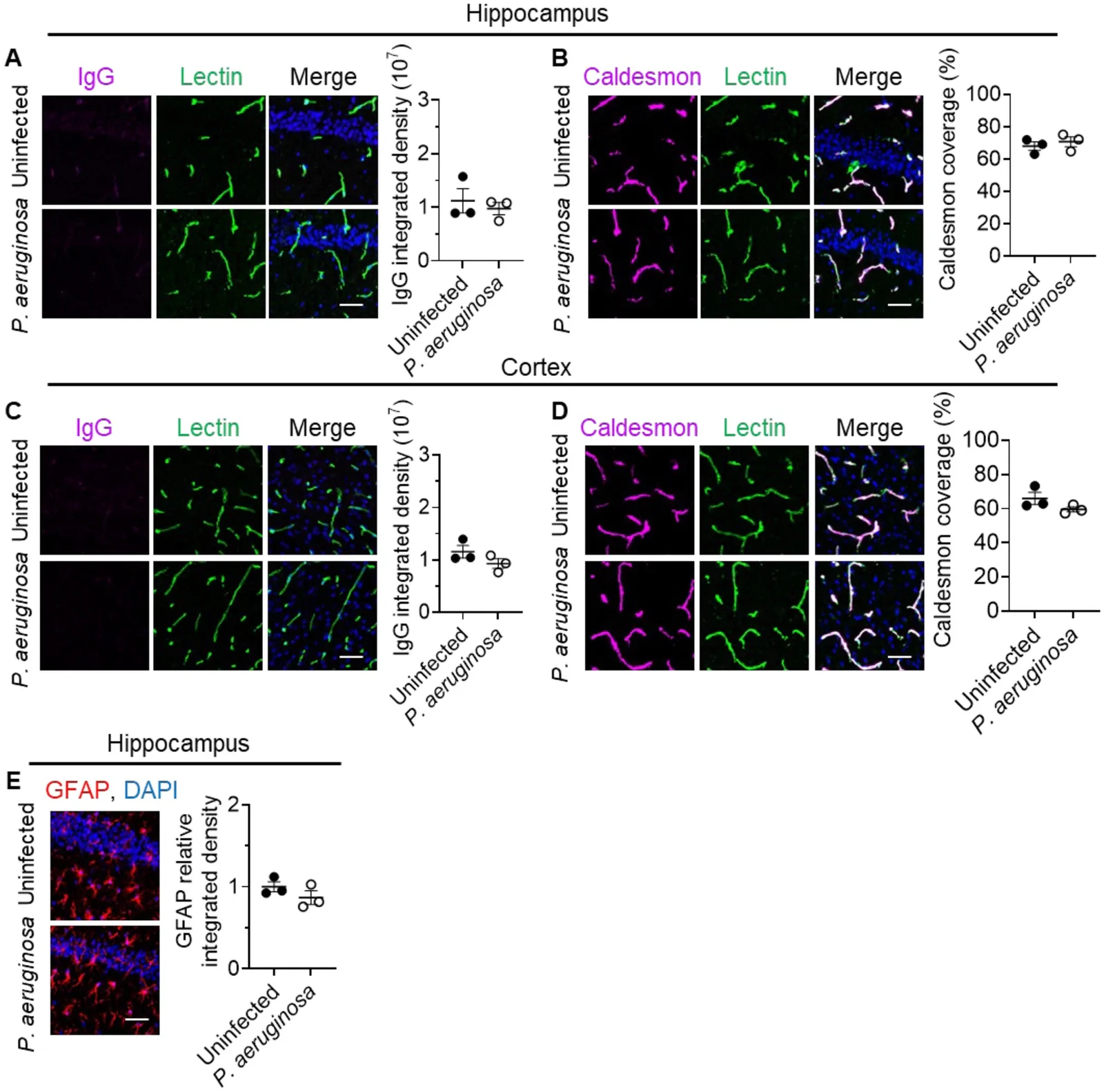
Sublethal *Pseudomonas aeruginosa* infection does not cause blood-brain barrier breakdown or neuroinflammation. (A, B) Representative images and quantification of integrated density of (A) IgG leakage from vessels (lectin) and (B) pericyte (caldesmon) coverage of vessels (lectin) as a percent change in the hippocampus. (C, D) Representative images and quantification of integrated density of (C) IgG leakage from vessels (lectin) and (D) pericyte (caldesmon) coverage of vessels (lectin) as a percent change in the cortex. (E) Representative images and quantification of integrated density of astrocytes (Glial Fibrillary Acidic Protein; GFAP) in hippocampus. The same set of slides were used to label caldesmon, IgG, lectin, and DAPI in 4 different fluorescent channels. This led to image duplication that is visible in panels A and B for lectin and DAPI for the *P. aeruginosa* infected group due to imaging the same areas. The representative images in this figure were cropped. The brightness/contrast of final images in PowerPoint were adjusted to increase visibility in the figures. No artificial data were added and no image signal was lost by the adjustments made. Data are shown as mean ± SEM with *n* = 3 mice per group. Statistical significance was assessed using a one-tailed t-test, where *P* < .05 was considered significant. None of the parameters assessed achieved statistical significance. Scale bar = 45 μm.

Severe pneumonia disrupts the blood-brain barrier and causes neuroinflammation.^37-39^ However, in the present studies of sublethal bacterial pneumonia in wild-type mice, where all infected animals survived, IgG (molecular weight, 150 kDa) translocation was not evident in either the hippocampus (Figure 2A) or cortex (Figure 2C), indicating that the blood-brain barrier integrity to large molecules remained intact. There was no change in pericyte (caldesmon) coverage of vessels in either the hippocampus (Figure 2B) or cortex (Figure 2D), and consistently, astrocytes were not activated, as determined by GFAP quantification (Figure 2E). Thus, the blood-brain barrier was not severely disrupted and overt neuroinflammation was not observed during sublethal *P. aeruginosa* bacterial pneumonia.

### Tau is necessary for *P aeruginosa* pneumonia to impair hippocampal information transfer

**Figure 3:**
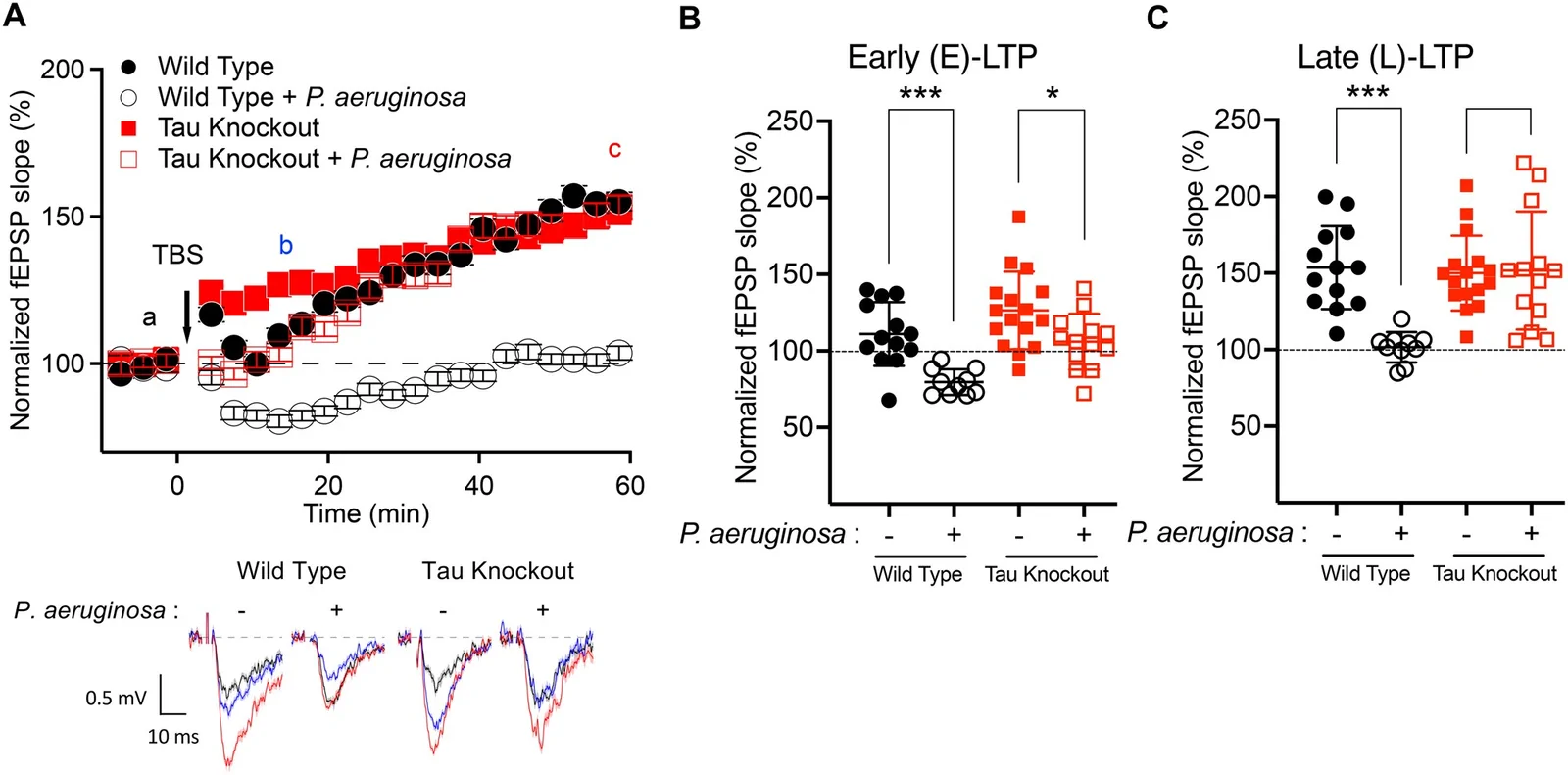
Tau is necessary for bacterial infection to suppress long-term potentiation in the hippocampus. The normalized field excitatory postsynaptic potential (fEPSP) was measured following theta burst stimulation (TBS) in the Schaffer collateral synapses as a surrogate of long-term potentiation (LTP). Representative traces are shown in (A) and summary data are highlighted in (B) and (C). (A) Hippocampi from wild-type and tau knockout animals were isolated under baseline conditions and 48 hours following intratracheal *Pseudomonas aeruginosa* infection. The normalized fEPSP is shown on the top panel. (a), (b), and (c) represent traces measured at baseline, 12-17 minutes following TBS, and 55-60 minutes following TBS, respectively; (b) represents the early (E) LTP; (c) represents the late (L) LTP. Examples of late LTP traces are shown in the bottom panel of (a). There was no difference in LTP in wild-type and tau knockout mice in uninfected controls. (A-C) Infection abolished both the early and late LTP in hippocampi from wild-type mice. Infection reduced early but not late LTP in hippocampi from tau knockout mice. ***Significant difference, *P*  <  .0005; *P  <  .05. Statistical differences were determined using 2-way ANOVA with multiple comparisons.

Prior studies have incriminated tau in the impaired hippocampal information transfer seen following infection,^18^^,^^21^^,^^22^ a principle that was further supported here. In wild-type animals, theta burst stimulation led to a time-dependent enhancement of the hippocampal fEPSP, indicative of learning (Figure 3A). Yet following sublethal infection, both the early and late hippocampal long-term potentiation were reduced (Figure 3B, C). In contrast, whereas early long-term potentiation was modestly reduced in hippocampi from tau knockout mice, the late long-term potentiation was preserved. These findings are consistent with the idea that cytopathic tau variants are necessary for sublethal infection to impair neural information processing.

### Rescue of 1N4R tau expression in lung capillary endothelium of tau knockout animals

**Figure 4:**
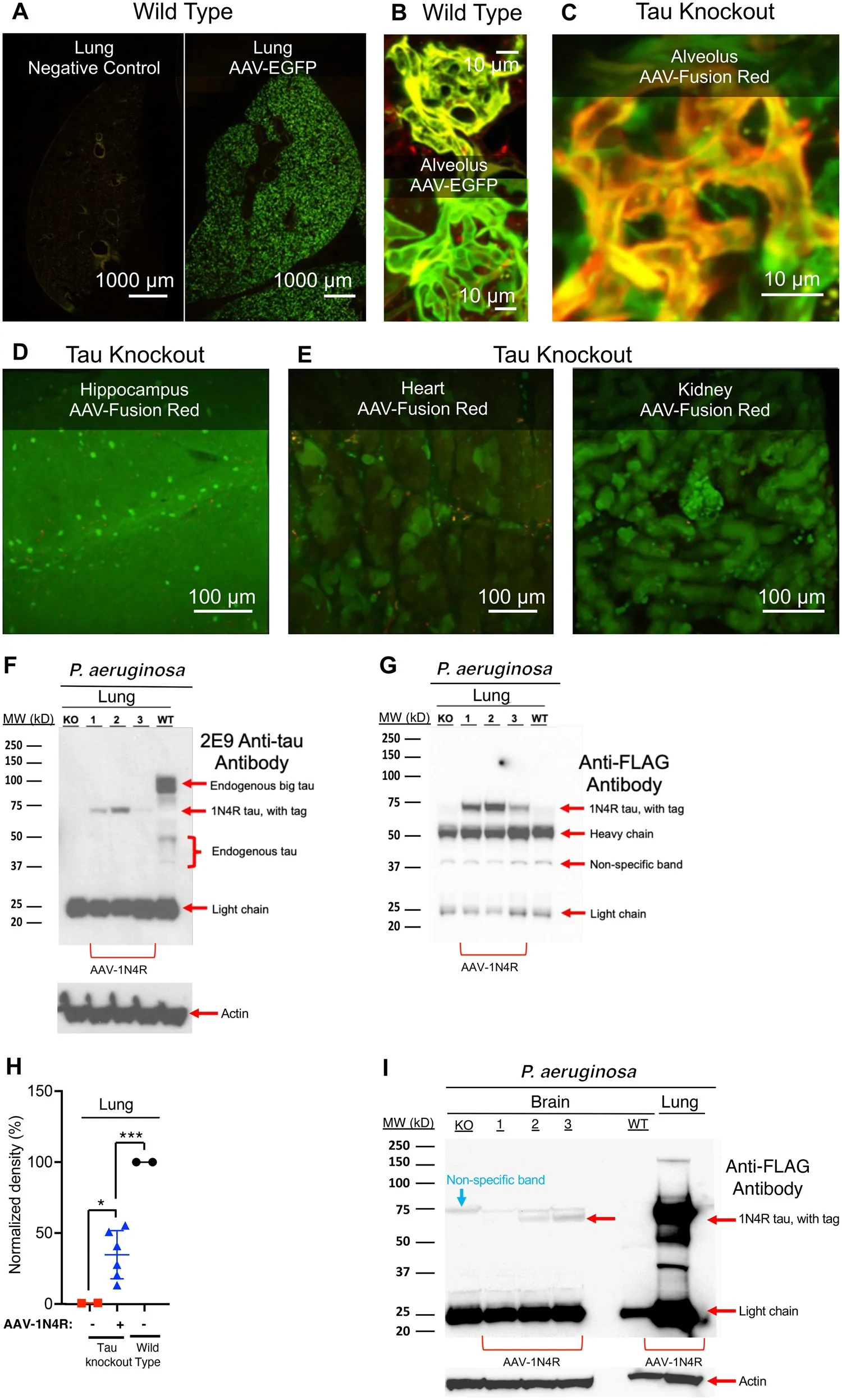
Gene delivery using an intronless AAV with endothelial tropism results in selective expression of reporter genes and 1N4R tau in lung capillaries. AAV was introduced by tail vein injection and imaging and western blotting performed 2-3 weeks later. Lung imaging was performed on thick precision lung slices (∼800 µm) following gelatin infusion into the circulation and agarose introduction into the airways, under physiological pressures. Imaging of hippocampus, heart, and kidney was performed on thick slices. (A) Negative control shows lung autofluorescence in in wild-type (WT) mice in comparison with AAV-delivered enhanced green fluorescent protein (EGFP). (B) High-power image shows examples of green fluorescence within the alveolar capillaries following AAV delivery of EGFP to WT mice. (C) High power image shows example of red fluorescence within the alveolar capillaries following AAV-delivery of Fusion Red to tau knockout (KO) mice. (D) Illustrates an absence of red fluorescence in the hippocampus following AAV delivery of Fusion Red to tau KO mice. (E) Illustrates an absence of red fluorescence in the heart and kidney following AAV delivery of Fusion Red to tau KO mice. (F) AAV-1N4R sustains lung tau expression 48 hours after *Pseudomonas aeruginosa* infection. Lungs from wild-type mice, but not from tau KO mice, express tau, as shown using the 2E9 anti-tau antibody. AAV-1N4R delivery results in 1N4R tau expression in the lungs of tau KO mice. (G) The blot in (F) was stripped and re-probed. AAV-mediated rescue of 1N4R tau expression in the lung was confirmed using the anti-FLAG antibody. (H) Relative tau expression was assessed by normalized densitometry. ***Significant difference, *P* <.0005; *P <.05. Statistical differences were determined using 2-way ANOVA with multiple comparisons. (I) After *P. aeruginosa* infection, a low 1N4R tau abundance was resolved in the brain of tau KO mice, as shown using the anti-FLAG antibody. Note that in this blot 80 µg of protein was loaded in the KO and AAV-1N4R lanes, whereas 8 µg and 20 µg of protein was loaded in the WT brain and lung lanes, respectively. A nonspecific band is seen. This nonspecific band is not seen in the WT lane because this lane was run with low protein. When the WT lane is run with higher protein levels, the nonspecific band is present (data not shown).

Since multiple nonneuronal cell types in the lung express tau, including lung capillary endothelia,^19^^,^^40-45^ we developed an approach to rescue tau expression solely in lung capillaries. AAV2 particles were packaged using a mutant AAV cap (see **Materials and Methods**), presenting a lung-targeting peptide (AAV2-ESGHGYF, referred to here as AAV), and they were employed to selectively express either EGFP, Fusion Red, or the 1N4R tau isoform in lung capillary endothelium.^28^ The expression of genes of interest in AAV was driven by either a CMV promoter (ie, intronless) or a CMV promoter plus the human beta-globin intron. The intronless AAVs were tested first. These AAVs were delivered to either wild-type mice or to global tau knockout mice (B6.129S4[Cg]-*Mapt*^tm1(EGFP/Klt^/J). The tau knockout mice used were notable because EGFP has been inserted into the tau locus so that any tau-expressing cell exhibits EGFP fluorescence.^19^^,^^21^ AAV-EGFP was therefore delivered to wild-type mice, whereas AAV-Fusion Red and AAV-1N4R tau were delivered to tau knockout mice. AAVs were introduced via the tail vein and 2 to 3 weeks later the lung, hippocampus, heart, and kidneys were prepared for analysis. Multiphoton imaging of precision lung slices revealed robust fluorescence of EGFP throughout the lung parenchyma (Figure 4A-C). High-power, 3-dimensional reconstruction of imaging illustrates that AAV transduced fluorescent proteins are expressed within the capillary network surrounding alveoli in both wild-type and tau knockout mice. The fluorescence pattern was uniform in capillaries, consistent with targeted expression in both general capillary and aerocyte endothelia. AAV reporter gene fluorescence was not detected in the hippocampus, heart, and kidney (Figure 4D, E). Thus, analysis of the intronless AAV-dependent reporter gene expression was indicative of selective capillary endothelial tropism. Studies testing gene expression using the CMV promoter plus the human beta-globin intron revealed robust gene expression, yet in some cases, gene expression was also detected in brain sections (data not shown); subsequent studies therefore utilized the intronless AAV to control tau expression.

Using the intronless AAV-1N4R, we tested for the presence of 1N4R tau following *P. aeruginosa* infection. Expression of AAV-delivered 1N4R tau was analyzed by western blotting in lung and brain (Figure 4F-I). The 2E9 anti-tau antibody was used to assess tau expression in the wild-type and tau knockout mice and in tau knockout mice receiving AAV-1N4R (Figure 4F). Tau was detected in the lungs of wild-type mice, and lung tau was not seen in the tau knockout mice. 1N4R tau was expressed in the lungs following delivery of the AAV-1N4R. These findings were corroborated using the anti-FLAG antibody (Figure 4G). Quantitative analysis of tau following AAV-1N4R delivery to the tau knockout mice revealed lung endothelium expressed approximately 35% of the normal lung tau abundance after infection (Figure 4H). A low level of 1N4R tau was detected in the brains of *P. aeruginosa*-infected tau knockout mice receiving AAV-1N4R (Figure 4I). Since delivery of the intronless AAVs led to detectable gene expression only in lung capillaries prior to bacterial infection, these data suggest that the low level of 1N4R resolved within the brain after infection originated in lung capillaries and translocated, likely via the circulation, to the brain.

### Lung capillary endothelial tau is a source of cytopathic tau following infection

**Figure 5:**
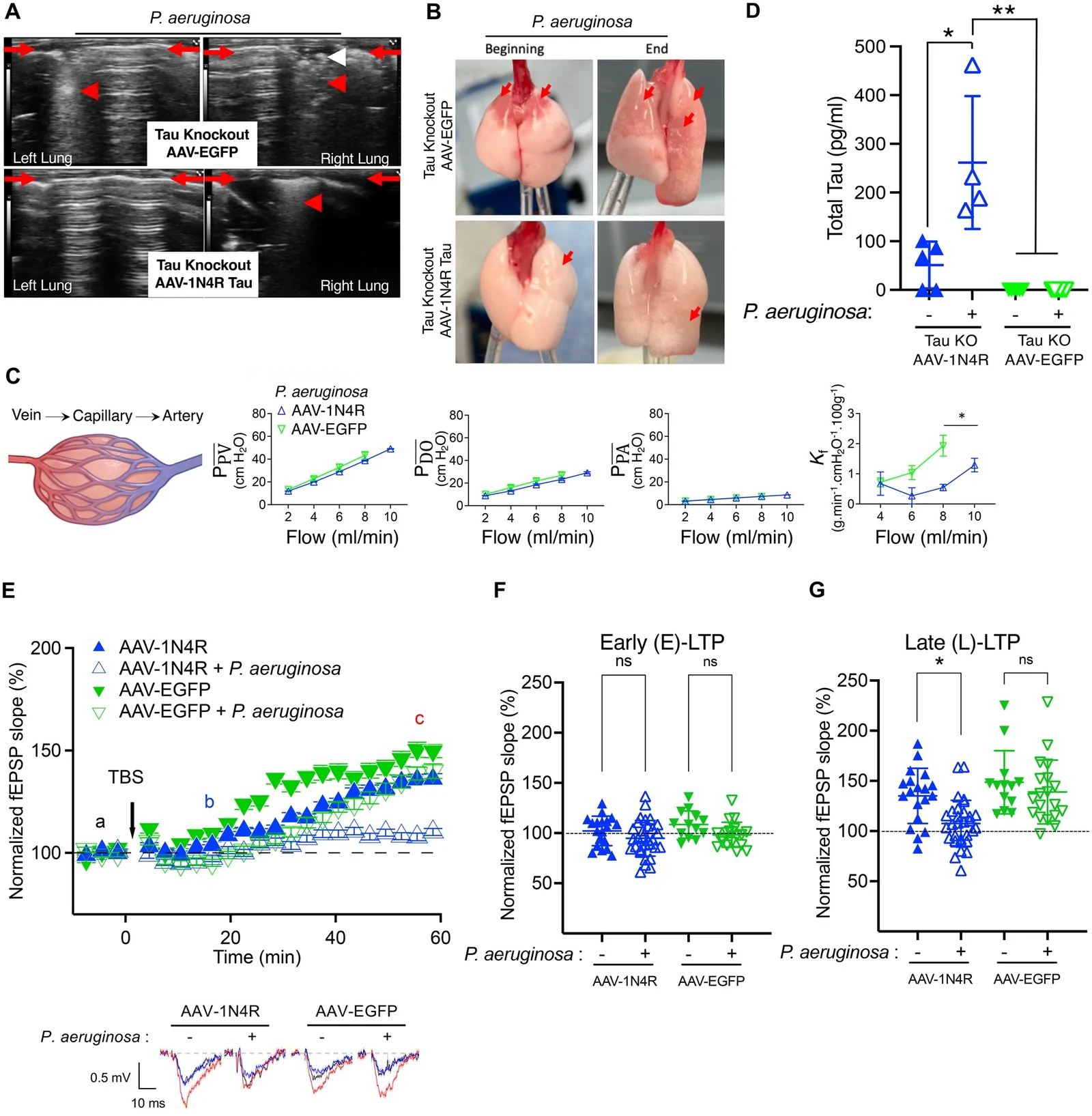
Lung endothelial 1N4R tau protects the alveolar-capillary membrane and is a source of circulating tau that inhibits long-term potentiation (LTP) following infection. (A) Intratracheal inoculation of *Pseudomonas aeruginosa* leads to a heterogeneous pattern of lung edema and consolidation 48 hours postinfection in tau knockout (KO) animals receiving either AAV-EGFP or AAV-1N4R tau. Representative ultrasound images of the left and right lung fields. Clear lung fields display A lines, characterized by hyperechoic regularly spaced horizontal repetitive lines, representing open and air-filled alveoli. Involved lung fields exhibit B lines, characterized by irregular focal hyperechoic opacities, representing fluid-filled airways with consolidation. Red arrows represent the pleural line, red arrowheads indicate B lines, and white arrowheads represent areas of consolidation. (B) Lung endothelial 1N4R tau expression improves alveolar-capillary barrier integrity following infection. Heart and lungs were removed en bloc. Lungs were ventilated and perfused over a range of flow rates (see also C). Representative lung images are shown following perfusion of lungs from *P. aeruginosa*-infected tau KO mice receiving either AAV-EGFP or AAV-1N4R tau. Lung edema was more severe in mice receiving AAV-EGFP than AAV-1N4R tau. (C) Lung endothelial 1N4R tau is an important determinant of the integrity of the alveolar-capillary barrier following infection. Perfusion rates were increased over time while pulmonary artery (P_PA_) and pulmonary vein (P_PV_) pressures were measured continuously and double occlusion (P_DO_) pressure was measured under stop-flow conditions. Permeability was assessed at each flow rate by filtration coefficient (K_f_). Although vascular pressures were similar in the lungs of tau KO mice receiving AAV-EGFP and AAV-1N4R tau, K_f_ was elevated in animals receiving AAV-EGFP. *n* = 3 and *n* = 4 mice in the AAV-EGFP and AAV-1N4R groups, respectively. (D) Lung endothelium contributes to circulating tau concentrations under baseline conditions and after infection. Circulating tau was detected in tau KO animals receiving AAV-1N4R tau, but it was not present in animals receiving AAV-EGFP. Circulating tau was further elevated in the AAV-1N4R tau–expressing mice after infection. (E) The normalized field excitatory postsynaptic potential (fEPSP) was measured following theta burst stimulation (TBS) in the Schaffer collateral synapses as a surrogate of LTP. Hippocampi from tau knockout animals expressing either EGFP (AAV-EGFP) or 1N4R tau (AAV-1N4R) following delivery of intronless AAVs to lung capillaries were isolated under baseline conditions and 48 hours following intratracheal *P. aeruginosa* infection. The normalized fEPSP is shown on the top panel. (a), (b), and (c) represent traces measured at baseline, 12-17 minutes following TBS, and 55-60 minutes following TBS, respectively; (b) represents the early (E) LTP; (c) represents the late (L) LTP. Examples of late long-term potentiation traces are shown in the bottom panel. Summary data are shown in panels (F, G). (F) There was no difference in E-LTP among any of the treatments. (G) L-LTP was reduced in the hippocampi of animals receiving AAV-1N4R but not in hippocampi of animals receiving the AAV-EGFP following infection. *P < .05; **P < .005; ns, not significant. Statistical differences were determined using 2-way ANOVA with multiple comparisons.

To test the importance of lung capillary endothelial tau in alveolar-capillary barrier integrity, either EGFP or 1N4R tau was expressed in lung capillaries of tau knockout animals using AAVs, and permeability was tested following *P. aeruginosa* pneumonia. Both EGFP- and 1N4R tau–expressing lungs exhibited evidence of pulmonary edema by ultrasound, including the presence of bilateral hyperechoic B-lines^18^ (Figure 5A). Gross morphological inspection of the lungs was consistent with injury to the alveolar-capillary membrane, although lungs expressing the 1N4R tau generally appeared to have less injury (Figure 5B). In isolated perfused lung studies, inflow, double occlusion, and outflow pressures were similar among EGFP- and 1N4R tau–expressing lungs, yet the capillary endothelial tau expression preserved alveolar-capillary barrier integrity (Figure 5C). Thus, capillary endothelial tau expression confers protection to the gas exchange barrier during infection, even though the presence of cytopathic tau variants within the airway and circulation can independently injure the lung.^18^^,^^19^^,^^46^^,^^47^

Prior studies revealed that tau is elevated in the circulation during ongoing infection,^48-50^ including bacterial pneumonia.^18^ The pulmonary circulation receives all of the cardiac output and lung capillary endothelium is in intimate contact with blood^19^; therefore, lung capillaries may contribute to the circulating pool of physiologic and cytopathic tau variants. AAV-mediated rescue of tau expression in lung capillaries was paralleled by detection of 1N4R tau in the circulations of 3 of 5 mice (Figure 5D). The circulating 1N4R tau was increased after infection and it was detected in all mice tested (Figure 5D), supporting evidence from prior studies that bacterial pneumonia promotes an elevation in cytopathic tau variants within the blood.^18^

Hippocampal long-term potentiation was assessed in the knockout animals expressing either EGFP or 1N4R tau in lung capillary endothelium (Figure 5E-G). Delivery of neither EGFP nor 1N4R tau AAVs to lung capillary endothelium negatively impacted long-term potentiation under baseline conditions. However, whereas AAV-mediated delivery of 1N4R tau did not decrease early long-term potentiation (Figure 5F), it reduced late long-term potentiation after infection (Figure 5G), similar to results seen in hippocampi from wild-type mice (Figure 3C). This result is in contrast to AAV-mediated delivery of EGFP, which neither impacted early nor late long-term potentiation. Thus, lung capillary endothelium is a source of tau that increases in the circulation and accesses the brain in conjunction with impaired hippocampal neural information processing during lower respiratory tract infection.

## Discussion

Delirium and incident dementia are commonplace in patients with lower respiratory tract infection, even in patients who are not hypoxemic and do not require ventilatory support.^1-10^ Tau has previously been incriminated as a mechanism responsible for impaired brain function during infection,^18-22^ yet important questions remain. In particular, cellular source(s) responsible for generation of the cytopathic tau that injures the lung and brain during lower respiratory infection remains controversial. Here, we examined whether lung capillary endothelial cell tau is important for alveolar-capillary barrier integrity, and whether lung endothelium generates cytopathic tau that contributes to brain injury during infection. The findings from this study revealed that lung capillary endothelial tau is an important determinant of alveolar-capillary barrier integrity during lower respiratory tract infection. Moreover, sublethal infection promoted lung capillary endothelial generation of cytopathic tau that was sufficient to disrupt hippocampal neural information processing, in the absence of severe blood-brain barrier breakdown.

### Lung tau and alveolar-capillary barrier integrity

Multiple nonneuronal cell types in the lung, kidney, liver, and pancreas express tau.^19^^,^^40-45^ Within the lung, tau is expressed in fibroblasts, pericytes, lymphatic endothelial cells, and capillary endothelial cells.^19^^,^^23^ Each of these cell types contribute to integrity of the alveolar-capillary membrane during infection and coordinately control parenchymal architecture to facilitate efficient gas exchange. Nonetheless, prior studies have not determined the contribution of lung tau on alveolar-capillary barrier integrity during infection. Our findings indicate that lung capillary endothelial tau is essential to maintaining and repairing the alveolar-capillary barrier during *P. aeruginosa* pneumonia. Four different lung tau isoforms have been identified, including the 0N4R, 1N4R, 2N4R, and Big Tau isoforms.^21^ Here, capillary endothelial expression of the 1N4R tau isoform was sufficient to rescue integrity of the alveolar-capillary niche during infection, and as such, represents at least one isoform implicated in regulation of the endothelial barrier.

While these studies reveal an essential role of the endogenously expressed lung capillary endothelial tau in alveolar-capillary barrier integrity, it is also evident that a proportion of endogenously expressed tau is hyperphosphorylated during infection, released from the cell, and subsequently recovered in the alveolus and from the circulation.^18^^,^^19^^,^^21^^,^^22^^,^^46^^,^^47^^,^^51^ The presence of cytopathic tau in the airway is strongly associated with lung injury.^47^ Moreover, circulating cytopathic tau disrupts the alveolar-capillary membrane, even in the absence of infection.^18^^,^^46^ Thus, whereas endogenously expressed lung endothelial tau fulfills an essential role in integrity of the alveolar-capillary barrier, the presence of hyperphosphorylated, cytopathic variants in the airway and circulation contributes to lung injury during infection.

### Lung tau disrupts neural information processing in the brain following infection

Tau is necessary for infection to impair hippocampal long-term potentiation.^18^^,^^20^^,^^22^^,^^46^ First, as shown previously^18^ and herein, infection inhibits hippocampal neural information processing in wild-type but not tau knockout mice. Second, infection-elicited cytopathic tau isolated from endothelium, lung tissue, and blood seed neuronal tau and impair long-term potentiation when incubated with hippocampi.^18^^,^^20^^,^^22^^,^^46^ A major unresolved issue is where the cytopathic tau variants originate during the natural course of infection. One prevailing idea is that lung infection leads to neuroinflammation that initiates tau pathology within the brain. There is precedence for this idea. Severe cases of pneumonia and sepsis can disrupt the blood-brain barrier, which allows access of circulating cytokines and inflammatory mediators into the CSF and brain parenchyma.^13^^,^^16^^,^^37-39^^,^^52-55^ Indeed, systemic inflammation disrupts the blood-brain barrier and provokes neuroinflammation,^56-58^ and neuroinflammation is a hallmark of tau pathology in Alzheimer’s disease and related dementias.^59-61^ Nonetheless, in the present studies sublethal bacterial pneumonia did not overtly disrupt the blood-brain barrier and enable passage of large molecular weight proteins (ie, 150 kD) into the parenchyma; it is notable that permeability to lower molecular weight proteins, similar to the size(s) of endogenous tau, was not tested. We did not observe overt neuroinflammation. Thus, our findings in this study are inconsistent with the idea that overt blood-brain barrier breakdown was the trigger for a neuronal inflammatory response severe enough to initiate localized tau pathology within the hippocampus.

An alternative possibility is that bacteria translocated into the brain, leading to localized neural tau hyperphosphorylation.^39^ Bacterial, parasite, fungal, and viral infections have been found in the brain in association with tau pathology,^62-69^ and recently, phosphorylated tau has been shown to possess antimicrobial activity.^70^ Hematogenous dissemination of *P. aeruginosa* pneumonia to the brain is an uncommon occurrence,^71-73^ although in cases of pneumonia, the bacteria that translocate into the brain need not be from the primary infecting organism.^39^ Pneumonia disrupts the alveolar-capillary barrier leading to dissemination of lung microbiome species via the circulation, which can access the brain leading to neuroinflammation.^39^ In this case, however, access of the infecting organism into the brain appears to require blood-brain barrier breakdown. The sublethal infection tested presently reveals that pneumonia can impair hippocampal neural information processing without overt blood-brain barrier breakdown or discernible evidence of neuroinflammation. These results are conceptually consistent with clinical evidence of brain dysfunction in ambulatory, nonhypoxemic patients.^2^

Here, we considered whether the pathogenic tau generated during lung infection can originate in tissues peripheral to the brain.^74^ To address this possibility, and more specifically to test the role of lung tau in this process, we designed studies in which tau expression was detected solely in lung capillaries. Remarkably, the expression of tau solely in the lung microcirculation was sufficient for infection to promote low-level tau accumulation within the brain coincident with impaired hippocampal long-term potentiation. Our findings provide strong evidence in support of the idea that cytopathic tau is generated in and released from lung capillaries during pneumonia, and further, that these variants circulate and can access the brain and trigger impaired neural information processing, even in the absence of blood-brain barrier breakdown. These findings do not exclude a role for neural tau in the natural course of infection or in response to circulating levels of cytopathic variants. Whereas cytopathic tau introduction into the circulation of wild-type mice abolishes hippocampal long-term potentiation, its introduction into the circulation of tau knockout mice reduces but does not eliminate long-term potentiation, suggesting that endogenously expressed tau is needed for cytopathic forms originating in the blood to fully impair neural information processing.^18^^,^^21^

### Limitations and future studies

The origin of circulating tau under physiological and pathophysiological conditions is incompletely understood. The prevailing dogma is that tau in the circulation mostly reflects neuronal activity.^75^^,^^76^ Tau is released from neurons.^77^^,^^78^ Extracellular neuronal tau accesses the interstitial fluid and is translocated into the CSF in an aquaporin-4–dependent manner, where it may be transported across the blood-brain barrier or cleared via the glymphatic system^79-81^ and/or through intramural peri-arterial drainage^82^ into the blood. These clearance mechanisms also pertain to disease states, as in Alzheimer’s disease and related dementias, where cytopathic tau is cleared from the brain via the glymphatic system^76^^,^^83^ or intramural peri-arterial drainage^82^ into the blood. Our studies add to this prevailing idea. Tau expressed solely in lung endothelium led to the appearance of tau in the blood, suggesting that lung endothelial tau may be released from capillaries into the circulation. Moreover, circulating tau concentrations were higher after infection concurrent with impaired hippocampal long-term potentiation. Thus, it is likely that lung capillaries contribute to the circulating pool of both physiologic and pathologic tau, although future studies are needed to trace endogenously expressed tau isoforms and resolve the proportion of circulating tau arising from each cellular source in order to comprehensively assess which tau isoforms are found in the blood under physiological and pathophysiological conditions.

Despite evidence that lung capillary endothelium is a source of tau in the circulation, and that infection elicits generation of cytopathic tau within the circulation that accesses the brain and impairs hippocampal function, the mechanism(s) by which blood-borne cytopathic tau enters the brain in the absence of overt blood-brain barrier disruption remain unknown.^19^ Tau proteins bidirectionally cross the blood-brain barrier.^84^ Both advanced glycosylation end product-specific receptor (AGER, formerly RAGE) and low-density lipoprotein receptor-related protein 1 (LRP-1) have been incriminated in tau transport across the blood-brain barrier and remain possible mechanisms.^19^^,^^85-88^ It is also possible that tau complexed with glycosaminoglycans or heparan sulfate fragments contributes to this translocation.^89-91^ Heparan sulfate proteoglycans facilitate tau uptake important for neuronal seeding.^89^ Given the evidence that sepsis causes endothelial cell glycocalyx shedding and release of 2-*O*- and *N*-sulfated heparan sulfate fragments that inhibit hippocampal long-term potentiation,^16^^,^^17^ future studies will consider the importance of heparan sulfate in tau transport and function within the brain.

Fate of the peripheral tau that has accessed the brain parenchyma, and any direct effects it may have on brain cells, is still poorly understood. Extracellular cytopathic tau may directly disrupt long-term potentiation by either altering presynaptic glutamate release or impairing postsynaptic spine morphology and density, both of which are required for long-term potentiation maintenance.^19^^,^^20^ Moreover, tau can be taken up by neurons, putatively via an LRP1-dependent mechanism, where it is capable of seeding endogenous tau and propagating between neurons, causing injury.^19^^,^^83^ Intracellular tau aggregates disrupt neuronal microtubule transport, which may negatively impact the translocation and insertion of ion channels and glutamate receptors into the membrane, thereby disrupting Ca^2   ^ homeostasis necessary for long-term potentiation.^84^ Pathological tau in astrocytes reduces glutamate uptake; excess glutamate escaping the synaptic cleft may lead to neuronal excitotoxicity. Circulating cytopathic tau may also interact with other neuropathic entities, including beta-amyloid and heparan sulfate, which are also generated following bacterial infection.^16^^,^^18^^,^^19^^,^^90^^,^^91^ Future studies are warranted to resolve how peripheral tau that accesses the brain interacts with neurons to impact neural information transfer.

It is also unclear how cytopathic tau variants relate to neuroinflammation. The current understanding is that neuroinflammation promotes tau pathology within the brain.^59-61^^,^^65^ Here, we show that cytopathic tau can originate in the lung, access the brain, and inhibit hippocampal long-term potentiation in the absence of overt blood-brain barrier breakdown and associated neuroinflammation. Moreover, emerging evidence indicates that tau is necessary for more severe bacterial infection to break down the blood-brain barrier and cause neuroinflammation; the link between circulating cytopathic tau variants and injury to the blood-brain barrier during the natural course of infection is under active investigation (Nelson, unpublished).

### Summary

Lung infection causes accumulation of cytopathic tau variants within the bronchoalveolar lavage fluid, lung, blood, CSF, and brain parenchyma.^18-22^^,^^46^^,^^47^^,^^51^ These variants exhibit characteristics of prion disease; they are heat-stable, protease-resistant, transmissible cytotoxins.^51^^,^^92^ They seed neural tau,^18^^,^^21^ providing evidence for a mechanism of injury propagation. The infection-elicited cytopathic tau variants are capable of injuring end-organs, including the lung and brain.^18-22^^,^^46^^,^^47^ Herein, we provide evidence that lung capillary endothelium is an important source of cytopathic tau during infection that is capable of injuring the brain. These findings support the idea that medical therapy targeting circulating cytopathic tau variants offers a tractable approach toward preserving brain function during infection and limiting progression of brain dysfunction following infection.

## Supplementary Material

aanag040_Supplementary_Data
