# Supplementary material for: Lung endothelial cytopathic tau is sufficient to impair long-term potentiation during infection
Source: Am J Respir Cell Mol Biol. 2026 Mar 10;74(8):1125–41. doi: 10.1093/ajrcmb/aanag040 (PMC13347185; doi:10.1093/ajrcmb/aanag040)
Supplement: aanag040_Supplementary_Data [file aanag040_supplementary_data.zip › AJRCMB Submission R1 FINAL Supplement - 2026.pdf]

Supplementary Information for

Lung endothelial cytopathic tau is sufficient to impair long-term potentiation during infection

Mike T. Lin *et al.*

Correspondence:

Mike T. Lin, Ph.D.
Phone: (251) 460-6816
Emails: mlin@southalabama.edu

Amy R. Nelson, Ph.D.
(251) 460-7004
amyreneenelson@usf.edu

Troy Stevens, Ph.D.
(251) 460-6056
tstevens@southalabama.edu

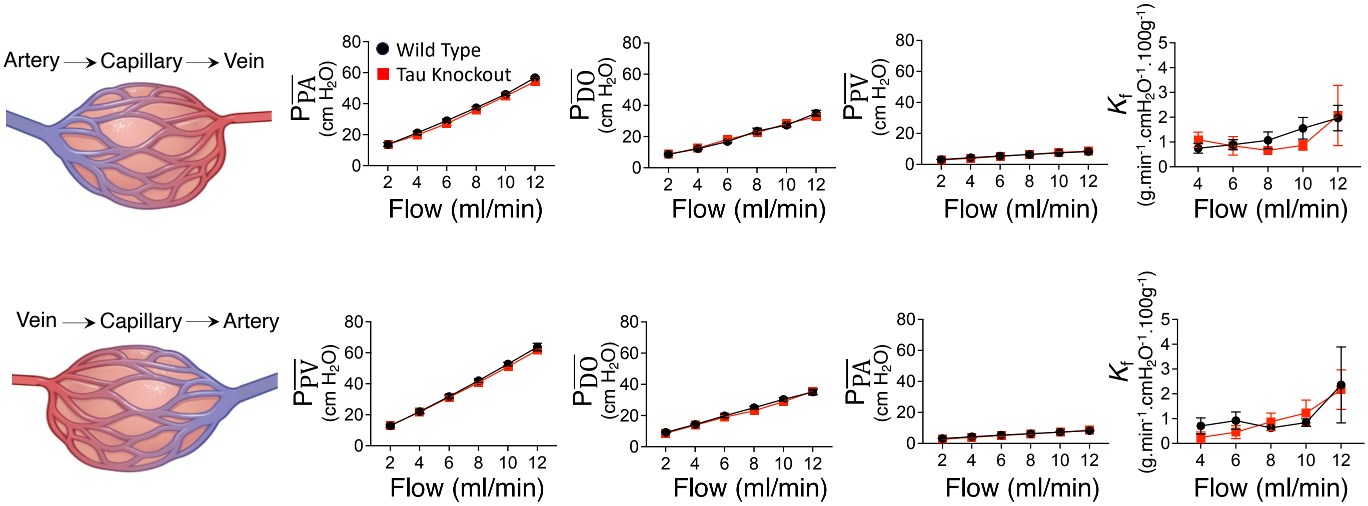

Figure S1. Lungs from wild type and tau knockout mice exhibit similar flow-pressure and permeability responses at baseline. Inflow (pulmonary artery, P_{PA}, or pulmonary vein, P_{PV}), double occlusion (P_{DO}), and outflow (pulmonary vein, P_{PV}, or pulmonary artery, P_{PA}) pressures were measured in the isolated perfused mouse lung. Permeability was estimated by measuring filtration coefficient, K_f. Flow-pressure responses and K_f are shown in forward [anterograde; (**A**)] and reverse [retrograde; (**B**)] flow orientations. Neither the flow-pressure nor K_f responses were significantly different (P = ns by two-way ANOVA and repeated measures) in the lungs when comparing wild type with tau knockout mice. Data are means $\pm$ SEM.

Primary Antibodies	Secondary Antibodies
BBB leakage	
Donkey anti-mouse IgG 568 (1:200); Invitrogen A10037	
Blood vessels	
	Lycopersicon Esculentum (Tomato) Lectin DyLight 649 (1:500); Vector Laboratories DL-1178 or Lycopersicon Esculentum (Tomato) Lectin DyLight 488 (1:500); Vector Laboratories DL-1174
Pericytes	
Rabbit anti-caldesmon XP (1:200); Cell Signaling 12503	Donkey anti-rabbit 568 or 647 (1:500); Invitrogen A10042 or A31573
Astrocytes	
Mouse anti-GFAP (1:500); Millipore Sigma MAB360	Donkey anti-mouse 568 (1:500); Invitrogen A10037

Table S1. List of reagents used to assess blood-brain barrier breakdown.
