# Supplementary material for: Lung endothelial cytopathic tau is sufficient to impair long-term potentiation during infection
Source: Am J Respir Cell Mol Biol. Author manuscript; Available in PMC 2026 Jul 10. (PMC13347185; doi:10.1093/ajrcmb/aanag040)
Supplement: Supplementary file 1 [file NIHMS2188123-supplement-FigureS1.pdf]

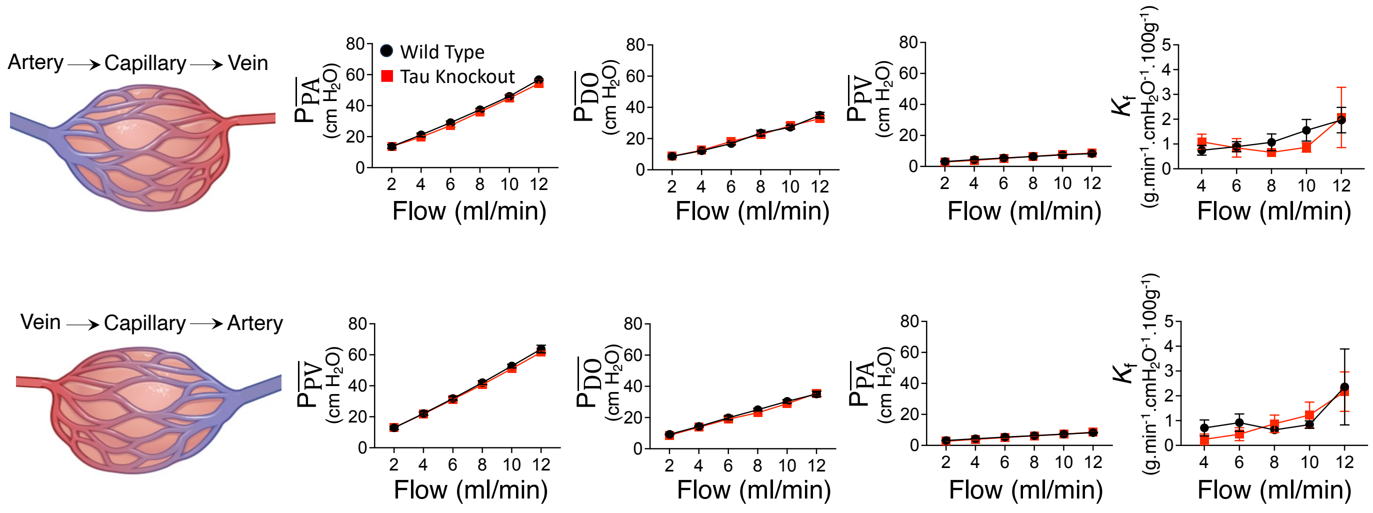

Figure S1. Lungs from wild type and tau knockout mice exhibit similar flow-pressure and permeability responses at baseline. Inflow (pulmonary artery, P_{PA}, or pulmonary vein, P_{PV}), double occlusion (P_{DO}), and outflow (pulmonary vein, P_{PV}, or pulmonary artery, P_{PA}) pressures were measured in the isolated perfused mouse lung. Permeability was estimated by measuring filtration coefficient, K_f. Flow-pressure responses and K_f are shown in forward [anterograde; (**A**)] and reverse [retrograde; (**B**)] flow orientations. Neither the flow-pressure nor K_f responses were significantly different (P = ns by two-way ANOVA and repeated measures) in the lungs when comparing wild type with tau knockout mice. Data are means $\pm$ SEM.
