# Supplementary material for: Lung endothelial cytopathic tau is sufficient to impair long-term potentiation during infection
Source: Am J Respir Cell Mol Biol. Author manuscript; Available in PMC 2026 Jul 10. (PMC13347185; doi:10.1093/ajrcmb/aanag040)
Supplement: Supplementary file 2 [file NIHMS2188123-supplement-TableS1.pdf]

Primary Antibodies	Secondary Antibodies
BBB leakage	
Donkey anti-mouse IgG 568 (1:200); Invitrogen A10037	
Blood vessels	
	Lycopersicon Esculentum (Tomato) Lectin DyLight 649 (1:500); Vector Laboratories DL-1178 or Lycopersicon Esculentum (Tomato) Lectin DyLight 488 (1:500); Vector Laboratories DL-1174
Pericytes	
Rabbit anti-caldesmon XP (1:200); Cell Signaling 12503	Donkey anti-rabbit 568 or 647 (1:500); Invitrogen A10042 or A31573
Astrocytes	
Mouse anti-GFAP (1:500); Millipore Sigma MAB360	Donkey anti-mouse 568 (1:500); Invitrogen A10037

Table S1. List of reagents used to assess blood-brain barrier breakdown.
